# Efficient production of a high-performance dispersion strengthened, multi-principal element alloy

**DOI:** 10.1038/s41598-020-66436-5

**Published:** 2020-06-15

**Authors:** T. M. Smith, A. C. Thompson, T. P. Gabb, C. L. Bowman, C. A. Kantzos

**Affiliations:** 1 NASA Glenn Research Center, Cleveland, OH 44135 USA; 2 Vantage Partners, 3000 Aerospace Pkwy, Brook Park, OH 44142 USA

**Keywords:** Mechanical properties, Metals and alloys, Design, synthesis and processing

## Abstract

Additive manufacturing currently facilitates new avenues for materials discovery that have not been fully explored. In this study we reveal how additive manufacturing can be leveraged to produce dispersion strengthened (DS), multi-principal element alloys (MPEA) without the use of traditional mechanical alloying or chemical reactions. This new processing technique employed resonant acoustic mixing to coat an equiatomic NiCoCr powder with nano-scale yttrium oxides. Then, through laser powder bed fusion (L-PBF), the coated powder was successfully consolidated into 99.9% dense parts. Microstructural analysis confirmed the successful incorporation and dispersion of nano-scale oxides throughout the build volume. Furthermore, high temperature mechanical testing of the DS alloys showed significant improvements in strength and ductility over the baseline NiCoCr. As a result, this recently discovered processing route opens a new alloy design and production path that is synergistic between additive manufacturing and dispersion strengthening, possibly enabling a new generation of high-performance alloys.

## Introduction

Additive manufacturing (AM) techniques have broadened many aspects of component design, enabled part count reduction, and decreased commissioning time for prospective NASA hardware and industrial applications^1^. Currently, the bulk of metallic AM research has been conducted on traditional alloys^2–4^. Yet, AM facilitates new avenues for materials discovery that have not been fully explored^5,6^. For laser powder bed fusion (L-PBF), the finely focused, laser melting of powder is analogous to welding and is thus problematic for alloys that are difficult to weld. Unfortunately, many of the alloys used for ultra-high temperature applications (>1000 °C) fall within this criterion. Therefore, there currently exists a need for high temperature alloys that can be produced through L-PBF or similar AM processes. One alloy system that has shown promise is the multi-principal element alloy (MPEA) class. The discovery and growth of this interesting new class of alloys, coined “High Entropy alloys”, has coincided with the development of AM. High entropy alloy development has led to the identification of a wide range of MPEAs, such as the ternary alloy NiCoCr, which has demonstrated impressive mechanical properties over a wide range of temperatures and stresses^7,8^. Recent studies have also presented favorable results from producing the NiCoCr alloy using AM^9^. The success of fabricating the “Cantor alloy” (NiCoCrMnFe) and its derivatives through AM may result from the small gap between their solidus and liquidus temperatures^10^, thereby, reducing the risk of heat affected zone (HAZ) cracking and lowering residual stresses^11^. Unfortunately, the phase simplicity that enhances the manufacturability of alloys such as NiCoCr will also limit their high temperature mechanical properties. An additional strengthening mechanism must be introduced, such as dispersion strengthening (DS).

Dispersion strengthening, primarily through the use of oxides, was a strengthening technique that was explored thoroughly in the 1980’s and was expected to replace existing Ni-base superalloys in extreme gas turbine environments^12^ and stainless steels in nuclear applications, such as advanced fission or fusion reactors^13,14^. Although an effective strengthening mechanism, the incorporation of nano-scale dispersoids into a metal matrix was found to be an extremely difficult manufacturing challenge that was cost prohibitive for many applications^15,16^. DS alloys typically were manufactured through a mechanical alloying (MA) process, in which the dispersoids were alloyed into the metallic powder through high energy ball milling. This process resulted in an alloy that was significantly more expensive to fabricate compared to more conventional superalloys^17^. Recent studies have successfully used AM to produce DS components^18,19^ and results on the mechanical properties of these alloys have been overall promising. However, a process such as mechanical alloying is still required to incorporate the dispersoids into the metal matrix prior to building with AM^20^. Furthermore, AM processing with mechanically alloyed powder is problematic because the highly deformed powders have poor flow and, thus, reduced feedstock delivery properties. Studies have also shown that powder shape and size distribution contribute significantly to the quality of AM builds^21^. Other methods of incorporating nanoparticles into additive powder lots through chemical reactions or depositions have been explored; though the added complexity and expense of these techniques may limit their commercial viability^15,17,22–26^.

For this study, we produced AM NiCoCr mechanical test specimens with dispersed nanoscale yttrium oxides (Y_2_O_3_) that are suitable for use in high-temperature applications through a new, economically viable method. Mechanical and microstructural analysis confirm the success this new fabrication pathway had in creating dispersion strengthened metallic alloys.

## Results

### Production of dispersion strengthened MPEAs via additive manufacturing

**Figure 1:**
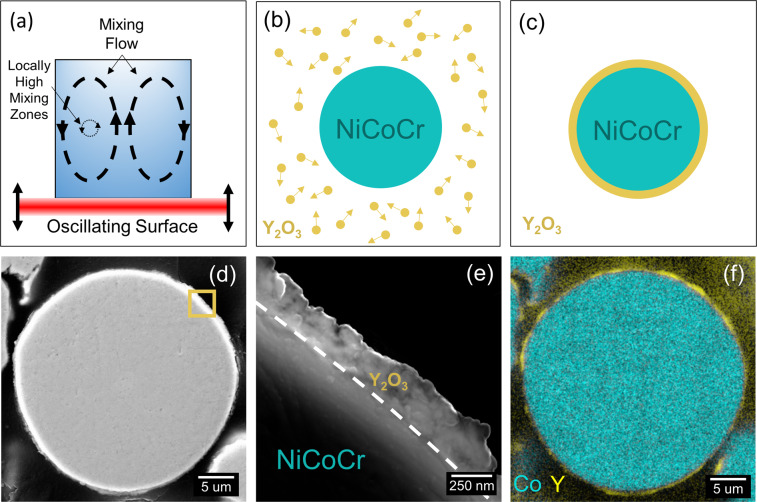
Acoustic mixing process used to produce Y_2_O_3_ coated NiCoCr powder. (**a**) A schematic demonstrating the acoustic mixing process. (**b**) Two powders were freely mixed for 1 hour resulting in (**c**) NiCoCr powder fully coated with a film of Y_2_O_3_ particles. (**d**) Scanning electron microscope (SEM) image of a coated NiCoCr powder (**e**) High resolution SEM image of the nano-scale Y_2_O_3_ film surrounding the NiCoCr particle. The area image is from the gold square highlighted in (**d**). (**f**) Composite chemical Co and Y chemical maps of the same particle in (**d**) confirming the Y_2_O_3_ film around the particle.

To produce AM material, equiatomic NiCoCr medium entropy alloy (MEA) powder which exhibited a diameter size range between 10–45μm and Y_2_O_3_ particles rated between 100–200 nm were acquired. A Resodyn LabRAM II resonant mixer was employed to coat the NiCoCr powder with one weight percent of nanoscale Y_2_O_3_^27–29^. This acoustic mixing process is illustrated in Fig. 1 along with images of the resulting powder.

**Figure 2:**
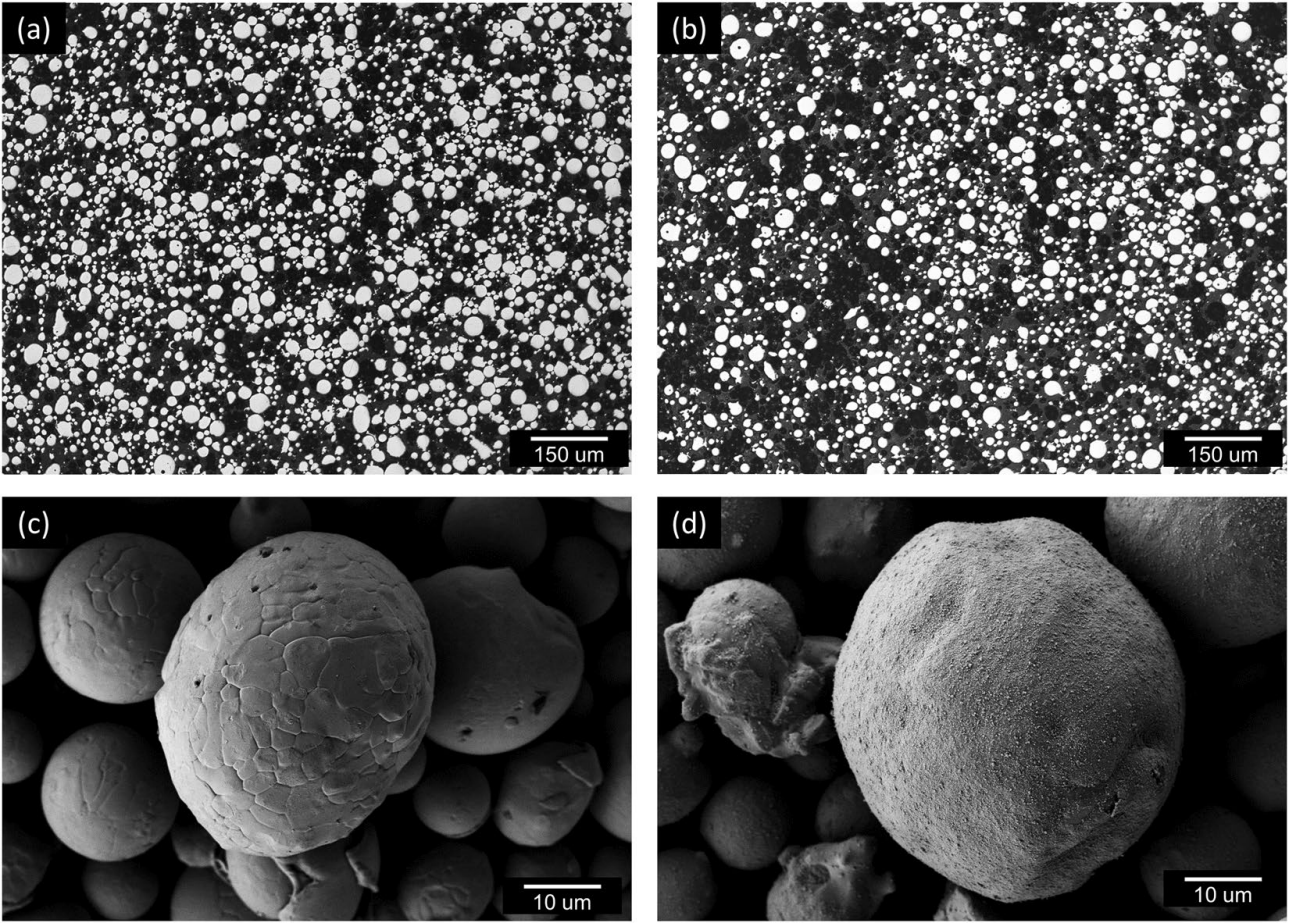
Comparison of pre- and post-mixed MPEA powder. (**a**) Optical image of the NiCoCr powder morphology before the acoustic mixing step with the Y_2_O_3_ nano particles and (**b**) after. (**c**) A high resolution SEM image of an uncoated NiCoCr particle from the same sample imaged in (**a**). (**d**) A high resolution SEM image of a coated NiCoCr particle from the same sample imaged in (**b**).

In Fig. 1(a), a diagram of the mixing mechanism is shown. Here, acoustic mixing employs a longitudinal pressure wave composed of a short amplitude and high frequency by attaining a resonance between a vibrating spring system and the stored mass of the powder and container^29^. This process quickly homogenized the powder (Fig. 1(b)), eventually coating the larger NiCoCr powder with a thin film of Y_2_O_3_ after an hour of mixing in a polyurethane container (Fig. 1(c)). A cross section of a coated powder particle imaged using a scanning electron microscope (SEM) and Everhart-Thornley detector is shown in Fig. 1(d,e). The high-resolution SEM image of the Y_2_O_3_ coating reveals the film to be roughly 250 nm thick. Energy dispersive spectroscopy (EDS) analysis confirmed that the particle is fully coated with the nano-scale Y_2_O_3_ as shown in Fig. 1(f). It is still not well understood how this Y_2_O_3_ coating is obtained and on-going work is being performed to better understand the underlying physics of this process. Qualitative Hall Meter flow tests indicated that the coated powder had similar flowability as compared to the virgin (V) uncoated NiCoCr powder. Optical and SEM microscopy revealed the morphologies of the NiCoCr powder to be unchanged after the mixing process in Fig. 2.

**Figure 3:**
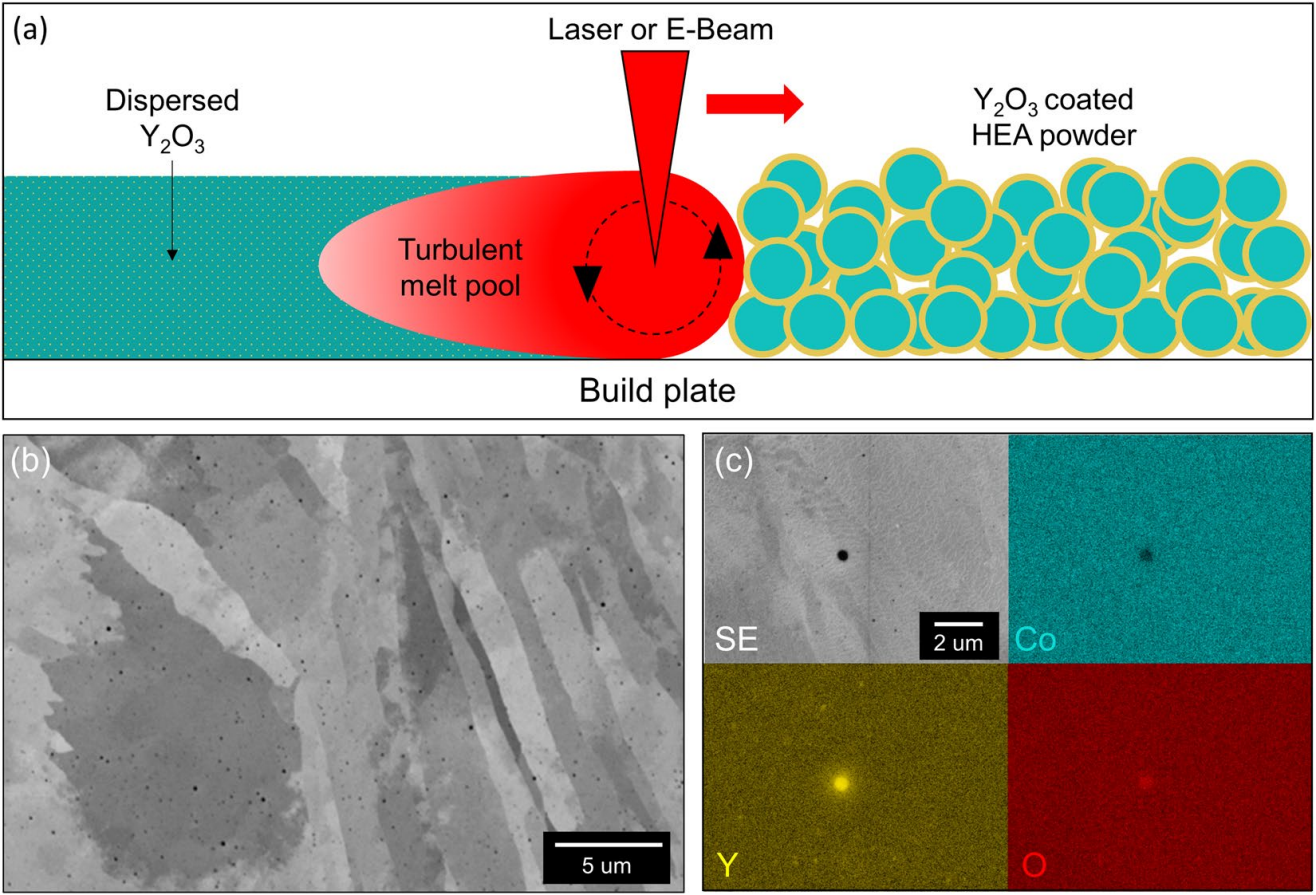
L-PBF production of a dispersion strengthened NiCoCr alloy. (**a**) A schematic demonstrating the incorporation of nano-scale Y_2_O_3_ particles into the NiCoCr AM build using the inherent turbulence of the meltpool. (**b**) High resolution SEM micrographs revealing a complete dispersion of oxides in the as-built microstructure. (**c**) Chemical maps confirmed that the dark particles shown in (**b**) are in fact the nanoscale Yttria particles.

Figure 2 also further confirms that the acoustic mixing step successfully coated the NiCoCr powder. In addition, the powder maintains its spherical morphology as compared to the highly deformed platelet-like powder produced through MA making it more suitable for AM. Baseline NiCoCr (V-MEA) and oxide dispersion strengthened NiCoCr (DS-MEA) mechanical and metallurgical test bars were successfully produced via L-PBF after a thorough exploration of the build parameter trade space. A recent study by Carnegie Mellon successfully captured the formation of a meltpool using L-PBF and the subsequent production of keyhole porosity. Their study successfully illustrated that the meltpool created by a laser during the L-PBF process is turbulent, mixing the liquid multiple times over before solidification can take place^30^. In fact, multiple studies have recently revealed that L-PBF melt pool turbulence can sufficiently disperse satellite ceramic particles into a metal matrix^18,23,24^. Figure 3(a) illustrates how it is believed this turbulent melt pool successfully dispersed the coated oxides into the metallic matrix of the build volume.

SEM micrographs of the dispersed oxides (Fig. 3(b)) confirm that the oxides are dispersed throughout the microstructure without any noticeable pattern that may have resulted from the laser scan path. EDS analysis in Fig. 3(c) confirms that the darker particles are Y_2_O_3_ and not some other contamination. Numerous other micrographs taken randomly throughout multiple builds reveal similar results, confirming the successful incorporation of the Y_2_O_3_ into the AM parts (Supplementary Fig. 1). The chemical maps in Fig. 3(c) also confirm that the NiCoCr remained a solid solution FCC phase during the L-PBF process. SEM analysis of the oxides using images represented in the Supplementary Fig. 1 found a 0.6% volume fraction of Y_2_O_3_ present in the as-built samples. This number should represent a lower bound of oxide content as some oxides may not be counted due to resolution limitations of the SEM. Separate build parameters were developed to produce the 99.9% dense parts for both the V-MEA and DS-MEA alloys. It should be noted that the energy density needed to produce fully dense builds using the coated powder was much higher than that needed for the uncoated feedstock. In fact, trying to use the DS-MEA build parameters on uncoated feedstock resulted in print failures due to the excessive energy input. Examples of the micrographs used to determine the porosity for both builds are displayed in Supplementary Fig. 2.

### Microstructural characterization of V-MEA and DS-MEA AM builds

**Figure 4:**
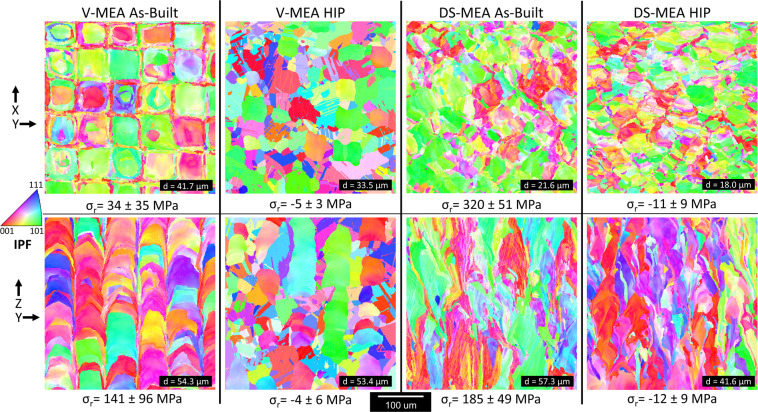
Microstructural analysis of V-MEA and DS-MEA before and after HIP. Electron back scatter diffraction (EBSD) inverse pole figure maps of the XY build plane and the YZ build plane where the Z-axis represents the build direction. Represented are maps from as-built and post-HIP samples without (V-MEA) and with Y_2_O_3_ (DS-MEA). Measured residual stress values of each sample are denoted by σ_r_ for each face. Error bars represent one standard deviation.

Following the successful L-PBF builds, the two alloys were characterized both before and after a high stress hot isostatic press (HIP) cycle at 1185 °C which was performed to relieve residual stress. The grain structures derived from electron back scatter diffraction (EBSD) maps of the V-MEA and DS-MEA builds with and without post processing are presented in Fig. 4 with the build direction denoted by the Z axis.

Large variations in grain structure and average grain diameter between both the as-built and post-HIP conditions for the V-MEA and DS-MEA specimen were observed. Average grain diameters (d) calculated from the EBSD maps are presented for each image. Interestingly, the laser path is evident in the as-built XY plane EBSD maps for both samples – producing a grid like grain structure of large grains surrounded by finer grains. For the case of the V-MEA builds, the HIP cycle promoted grain recrystallization and growth resulting in a much more equiaxed structure though some large elongated grains were still captured in the ZY plane EBSD maps. In contrast, the Y_2_O_3_ in the DS-MEA specimen clearly suppressed grain growth and recrystallization by pinning the grain boundaries both during the L-PBF build process and subsequent HIP cycle. Indeed, minimal recrystallization was observed in the post-HIP DS-MEA specimen, retaining the grain texture and finer average grain size, as compared to the V-MEA builds. Twin formation was also suppressed in the DS-MEA samples. Despite the minimal grain boundary movement during the HIP cycle for the DS-MEA sample, XRD analysis revealed that the residual stress (σ_r_) was successfully reduced from>300 MPa to near zero. SEM-EDS and X-ray diffraction confirmed that the NiCoCr MEA matrix remained a disordered solid solution in every stage of processing explored (Supplementary Fig. 3).

### Mechanical testing of AM samples

**Figure 5:**
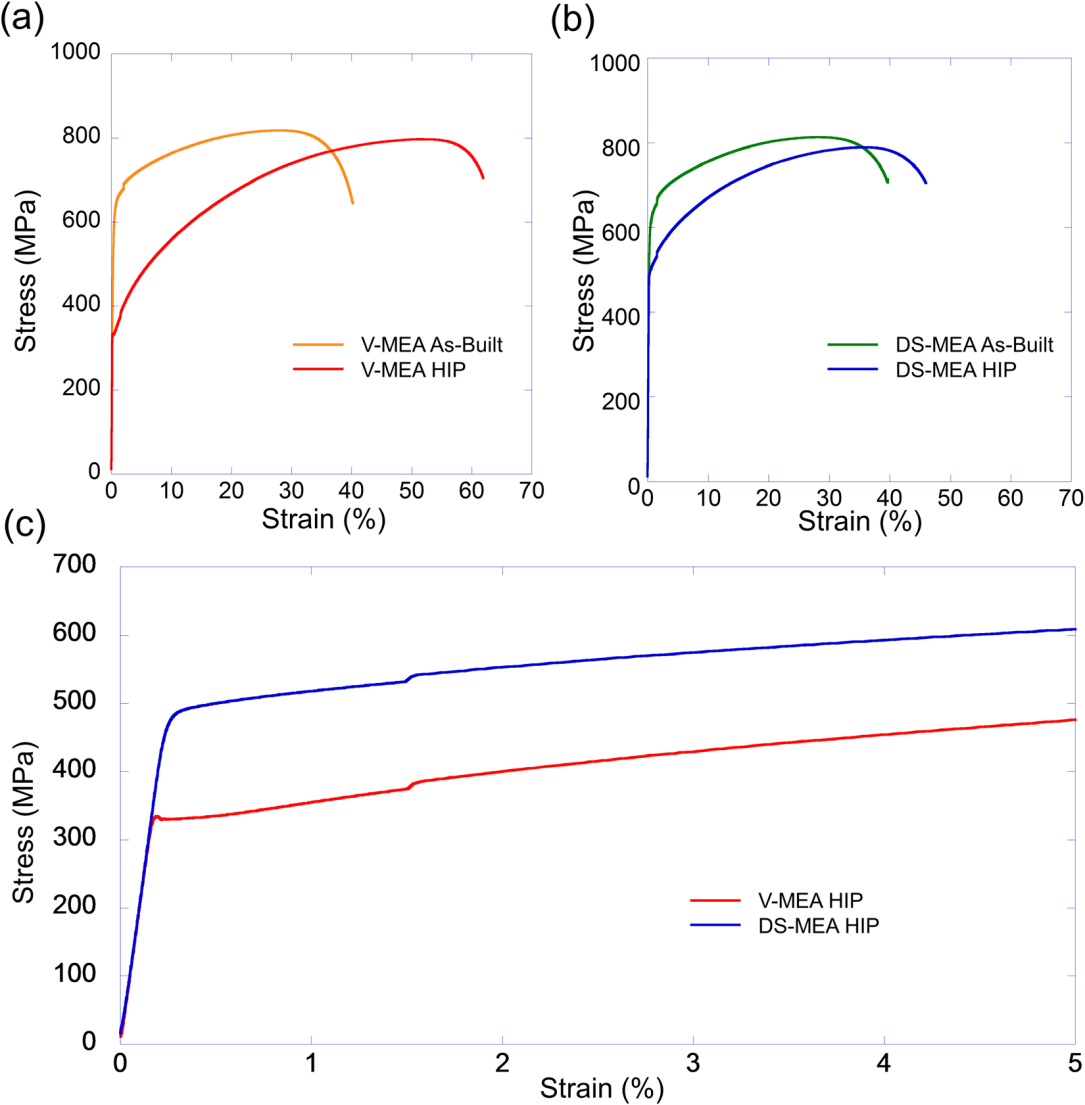
Mechanical tests of V-MEA and DS-MEA specimen. Room temperature tensile curves for the as-built and post-HIP conditions of the (**a**) V-MEA and (**b**) DS-MEA specimen. (**c**) Yield strength comparison between the V-MEA HIP and DS-MEA HIP specimen. The kink between 1–2% strain resulted from an increase in the tensile strain rate which is consistent with ASTM E8 standard.

All four sample conditions that were analyzed in Fig. 4 were subsequently tensile tested at room and 1093 °C according to the ASTM standards E8 and E21. Figure 5 presents the stress-strain curves from the room temperature tensile tests.

**Figure 6:**
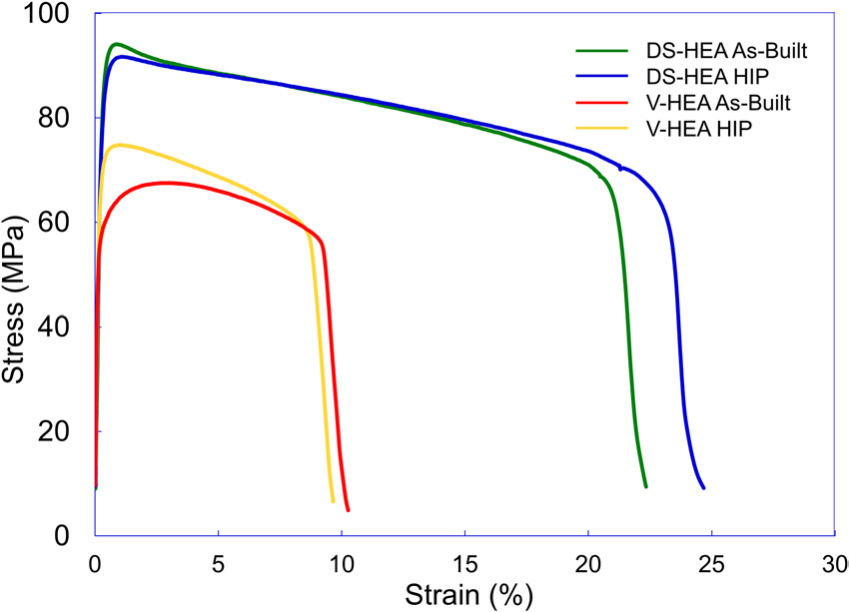
High Temperature properties of V-MEA and DS-MEA specimen. 1093 °C tensile curves for as-built and HIP V-MEA and DS-MEA alloys.

Notably, Fig. 5(c) reveals the post-HIP yield strength of the DS-MEA to be 50% higher than the V-MEA sample (496.4 MPa vs 331.6 MPa) further confirming the successful production of a dispersion-strengthened alloy. The DS-MEA specimen also retained the pronounced elongation and strain hardening properties inherent in the NiCoCr MPEA^31^. In addition, the strength and ductility exhibited by the HIPed V-MEA specimen is comparable to conventionally produced NiCoCr alloys while the finer grain structure in the as-built V-MEA sample provides higher strength and less ductility^8,32^. Figure 6 reveals the 1093 °C tensile test results.

Figure 6 reveals that the DS-MEA alloys possessed notably improved high temperature properties over the baseline NiCoCr specimen. In the as-built condition, the inclusion of the Y_2_O_3_ nano particles provided a > 35% increase in ultimate strength and >2.5x improvement in ductility compared to the V-MEA sample. Consistent improvements were found for the HIP specimen as well. Fracture surfaces from the elevated temperature tests were examined to better understand the large strength and ductile differences between the V-MEA and DS-MEA specimen. Fractography analysis reveals tensile failure, with some evidence of oxidation damage. The DS samples displayed significantly more ductility as seen in Supplementary Fig. 4. This increase in ductility may be due to the Yttria dispersions in the DS specimen maintaining a finer grain structure compared to the baseline NiCoCr samples. The baseline samples also appeared to be more susceptible to localized oxidation damage. Future work aims to further explore these failure differences and better understand the effects the nano-scale dispersions have on different high temperature properties, such as creep.

## Discussion

### Comparison to present day state of the art high temperature materials

**Figure 7:**
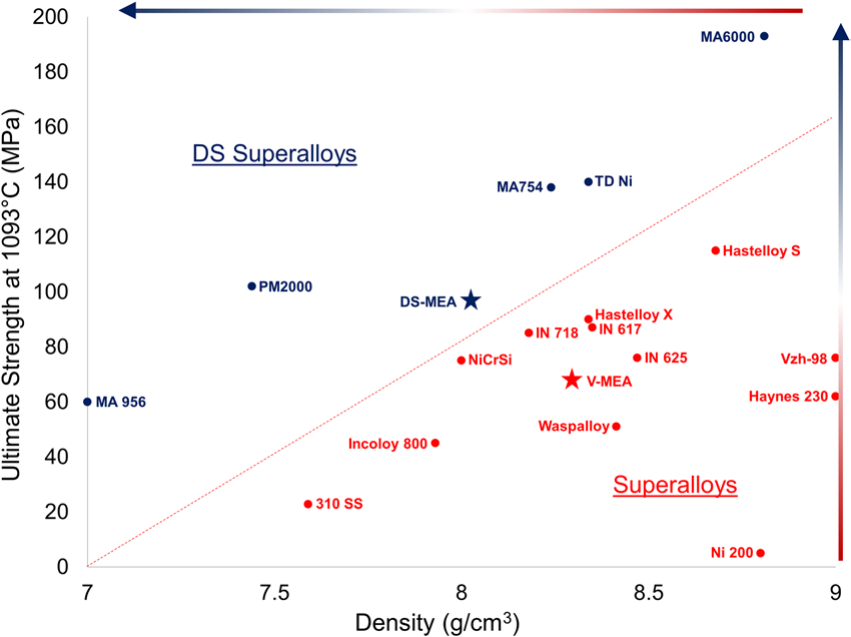
Ultimate tensile strength vs. density comparison between dispersion strengthened and wrought superalloys^33–38^. Scatter plot of alloy density and ultimate tensile strength at 1093 °C for conventional and dispersion strengthened alloys. V-MEA and DS-MEA are denoted by star points. Arrows highlight improvements in strength and density.

Figures 5 and 6 revealed that the DS-MEA specimen exhibited superior mechanical properties over the baseline V-MEA samples. This was most evident at the higher temperature were the oxides provided significant improvements in strength and ductility. To better understand how these properties compare to the present day state-of-the-art wrought and DS superalloys, the 1093 °C ultimate strength vs. density of these alloys are plotted together in Fig. 7^33–38^.

The plot in Fig. 7 compares the high temperature properties of both DS superalloys (blue), which are currently quite difficult and resource intensive to produce, and conventional wrought superalloys (red). The DS-superalloys clearly present a strength improvement compared to similarly dense superalloys. Most notably is the finding that the DS-MEA specimen presents similar properties as other DS-superalloys. Indeed, compared to the wrought superalloys, only Hastelloy S presented a higher ultimate strength than the DS-MEA specimen. This finding confirms that this new DS manufacturing process can be successfully leveraged to produce alloys with comparable high temperature properties to current DS-Superalloys. These findings are even more impressive considering that many aspects of the DS-MEA production have not yet been optimized, including oxide amount, grain structure, or heat treatment.

In conclusion, we present for the first time the ability to produce DS alloys through AM processes without requiring resource intensive steps, such as mechanical alloying, to incorporate dispersoids. The resulting DS material exhibited a 50% increase in room temperature yield strength over the baseline alloy with both samples in the HIP’ed condition. More notably, the addition of nanoscale dispersed Y_2_O_3_ in the NiCoCr improved its high temperature ultimate strength by >35% and increased its ductility almost three-fold over the baseline NiCoCr. This economical fabrication technique represents a new, unexplored approach to producing high temperature and high strength materials that until now have been either difficult or impossible to fabricate.

## Methods

### Materials

NiCoCr powder was purchased from Praxair Inc. The powder’s composition in weight percent was 34.66% Cobalt, 30.29% Chromium, 34.90 Nickel, combined with trace amounts (<0.02%) of Silicon, Iron, Nitrogen, and Oxygen. The powder was sieved using +270 and −325 mesh to acquire an average diameter of 14.1um as determined using a Horiba PSA300 Static Image Analysis System Particle Size. The dispersoid used in the AM process was nanoscale Y_2_O_3_ powder acquired from American Elements with a diameter range between 100–200 nm. The powder was certified 99.999% pure Yttrium Oxide.

#### Dispersion strengthened AM process

Batches comprising of 500 g of NiCoCr and 5 g of Y_2_O_3_ powder were poured together in a polyeruthane container and sealed using electric tape around the lid. Each container was mixed using a Resodyn LabRAM II Acoustic Mixer in 10-minute increments for a total of 60 minutes, letting the powder cool between each session. Post-mixed powder was then sieved using a 230 mesh screen to remove any large oxide or metallic powder particles. Both unmixed (V-MEA) and mixed (DS-MEA) NiCoCr powders were used to additively build microstructural and mechanical test components using an EOS M100 selective laser melting machine. Two-inch tall vertical test specimens were built upon 304 stainless steel build plates. All samples were then removed from the build plates using electric discharge machining (EDM).

### Microstructural characterization

For SEM analysis, samples were polished using SiC grit paper followed by 0.5 diamond suspension. Afterwards a final polish using 0.05 colloidal silica for 24 hours was employed on samples used for EBSD analysis. Oxide volume fraction analysis was performed on a Zeiss Auriga-FIB using an Everhart-Thornley secondary electron detector with low accelerating voltage (3 kV). By utilizing low accelerating voltage sub-surface oxide particles were avoided, thereby ensuring a more accurate volume fraction measurement. EBSD orientation mapping was performed using an EDAX Hikari EBSD detector with an 800 nm spot size. Post-processing of the maps was done using the TSL OIM Data Collection 7 software. Average grain diameters extracted from the maps did not include twin boundaries or grain sizes less than 3 um to remove effects from scan noise. High resolution imaging of the Y_2_O_3_ coating on the NiCoCr powder was performed using a Tescan MAIA3 in the ultra-high resolution (UHR) configuration at 15 kV. Chemical maps were performed using an Oxford Ultim Max Silicon Drift Detector and Aztec Software. Residual stresses were measured at the surface using a Bruker D8 Discover (area detector) X-ray diffractometer aligned in accordance with the approach and error bounds specified in ASTM E 915–10 but applied to the side-inclination rather than iso-inclination method. Data was gathered using Mn Ka radiation and the (311) crystallographic plane on a specimen target area of 1.2 mm^2^. Each residual stress dataset consisted of 24 area detector frames taken at 4 sample tilt (psi) angles (0°, 15°, 30°, and 45°) and 6 sample rotation (phi) angles (0°, 45°, 90°, 180°, 225°, and 270°). X-ray penetration depth decreased with increasing psi angle, going from 29 μm to 20 μm, representing depths that correspond to a 99 percent contribution to the diffracted beam. These X-ray results were analyzed using the Bruker LEPTOS v.7 software. Peak width was measured using the TOPAS program.

### Specimen processing after AM

To better understand the effect post-processing may have on both the microstructure and mechanical properties of the L-PBF MEA samples different a HIP cycle was performed. Samples of both the V-MEA and DS-MEA underwent a HIP cycle at 1185 °C while wrapped in Ta foil to mitigate oxidation. The HIP cycle also had the benefit of removing residual stress. This provides a better comparison between the DS and V-MEA samples as residual stress has been shown to affect mechanical properties^39^.

### Mechanical testing

Room temperature and elevated tensile tests were performed by Metcut Research Inc. for both the V-MEA and DS-MEA test specimen after different post processing pathways. All tensile tests were performed using ASTM E8 standard.

## Supplementary information

Supplementary information.

## Data Availability

The data that support the findings of this study are available from the corresponding author on reasonable request.
