# Supplementary material for: Efficient production of a high-performance dispersion strengthened, multi-principal element alloy
Source: Sci Rep. 2020 Jun 15;10:9663. doi: 10.1038/s41598-020-66436-5 (PMC7296023; doi:10.1038/s41598-020-66436-5)
Supplement: Supplementary file 1 — Supplementary information. [file 41598_2020_66436_MOESM1_ESM.docx]

Efficient production of a high-performance dispersion strengthened, multi-principal element alloy

T.M. Smith*^1^, A.C. Thompson^2^, T.P. Gabb^1^, C.L. Bowman^1^, C.A. Kantzos^1^

^1^NASA Glenn Research Center, Cleveland OH 44135 USA

^2^Vantage Partners, 3000 Aerospace Pkwy, Brook Park, OH 44142, USA

*email: timothy.m.smith@nasa.gov

**
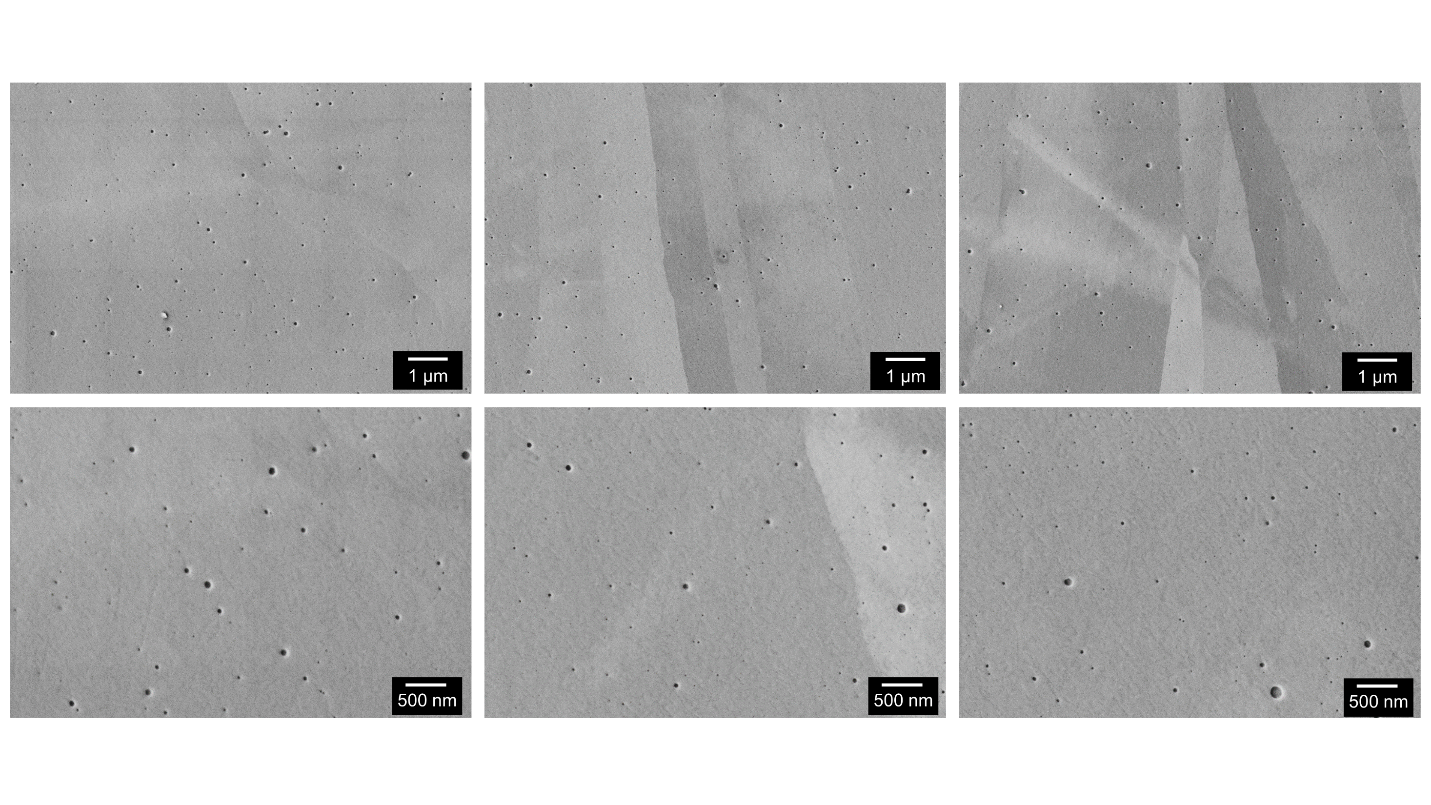
**

**Supplementary Fig. 1: High resolution SEM micrographs of dispersed oxides.** Low keV SEM secondary electron micrographs taken randomly throughout the DS-MEA as-built microstructure. The dark spherical particles were found to be dispersed Y_2_O_3_ as shown in Figure 2.


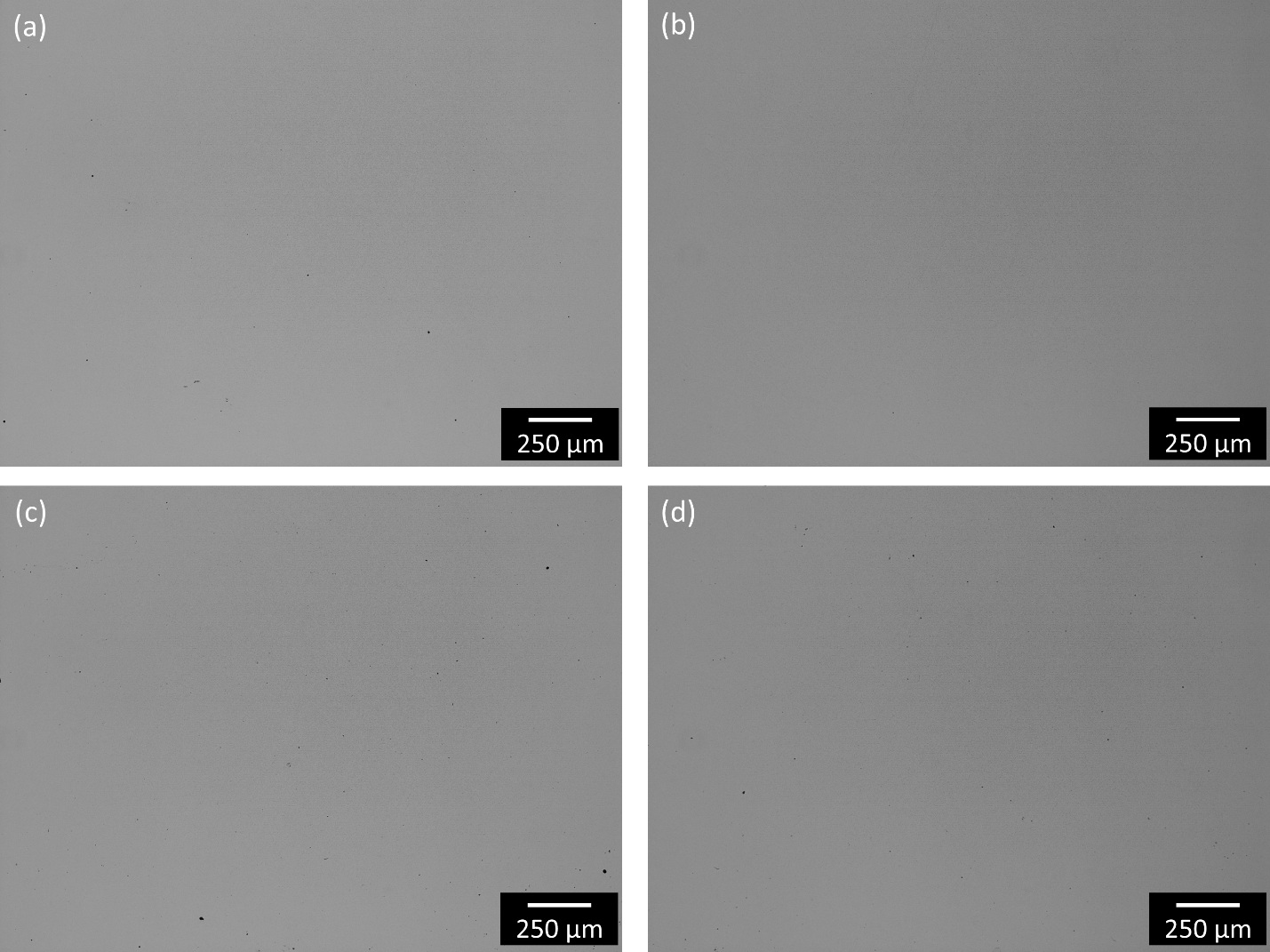


**Supplementary Fig. 2: Optical Micrographs of virgin and DS builds.** Representative optical micrographs of the porosity (Black areas) in the (a) As-built V-MEA, (b) post-HIP V-MEA, (c) As-built DS-MEA, and (d) post-Hip DS-MEA specimen.


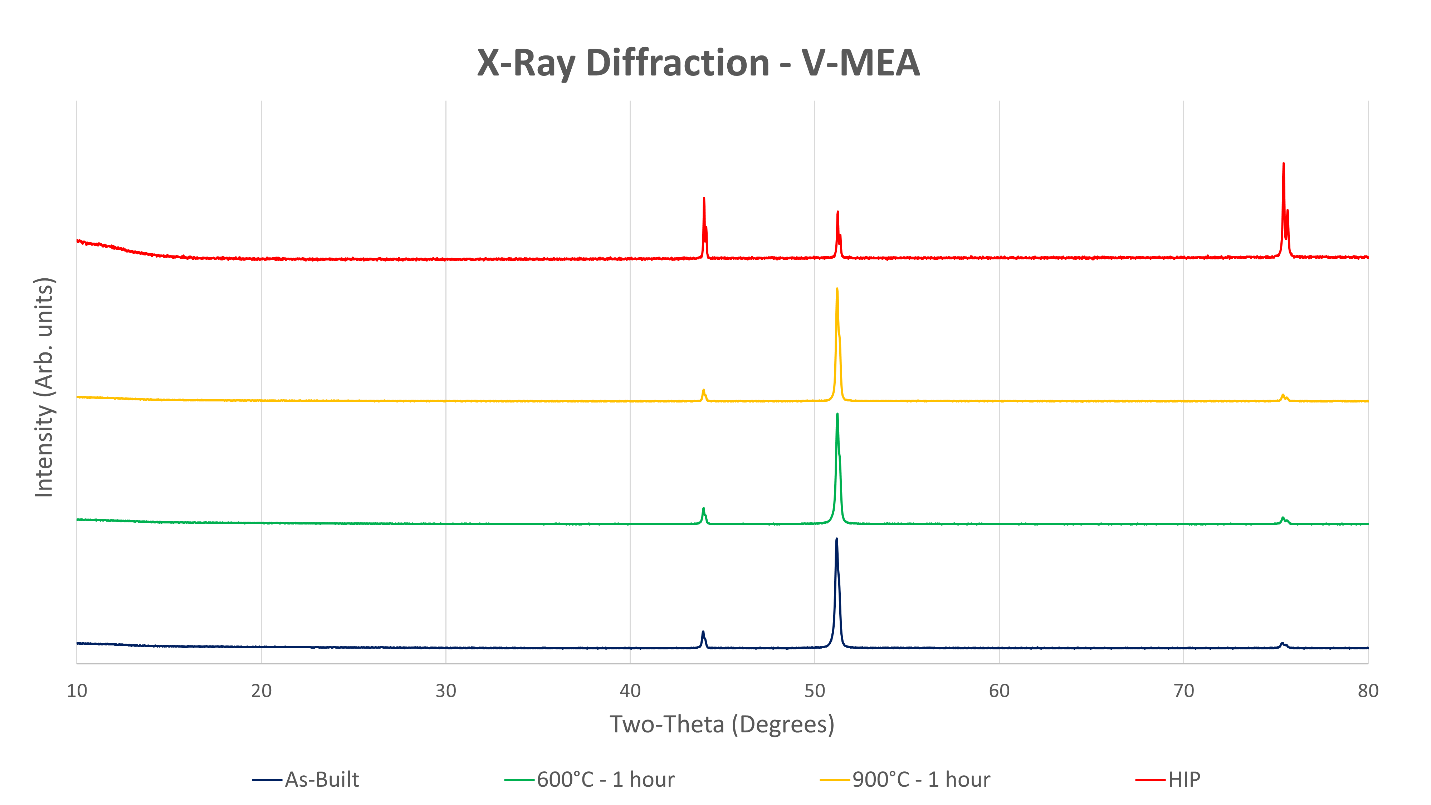


**Supplementary Fig. 3**: **X-ray diffraction phase identification.** X-ray diffraction results revealing FCC solid solution NiCoCr after varying post processing pathways. Peak intensity differences observed in the HIP spectrum are due to grain texture changes that occurred during the HIP process. In this case the grain structure became more equiaxed.


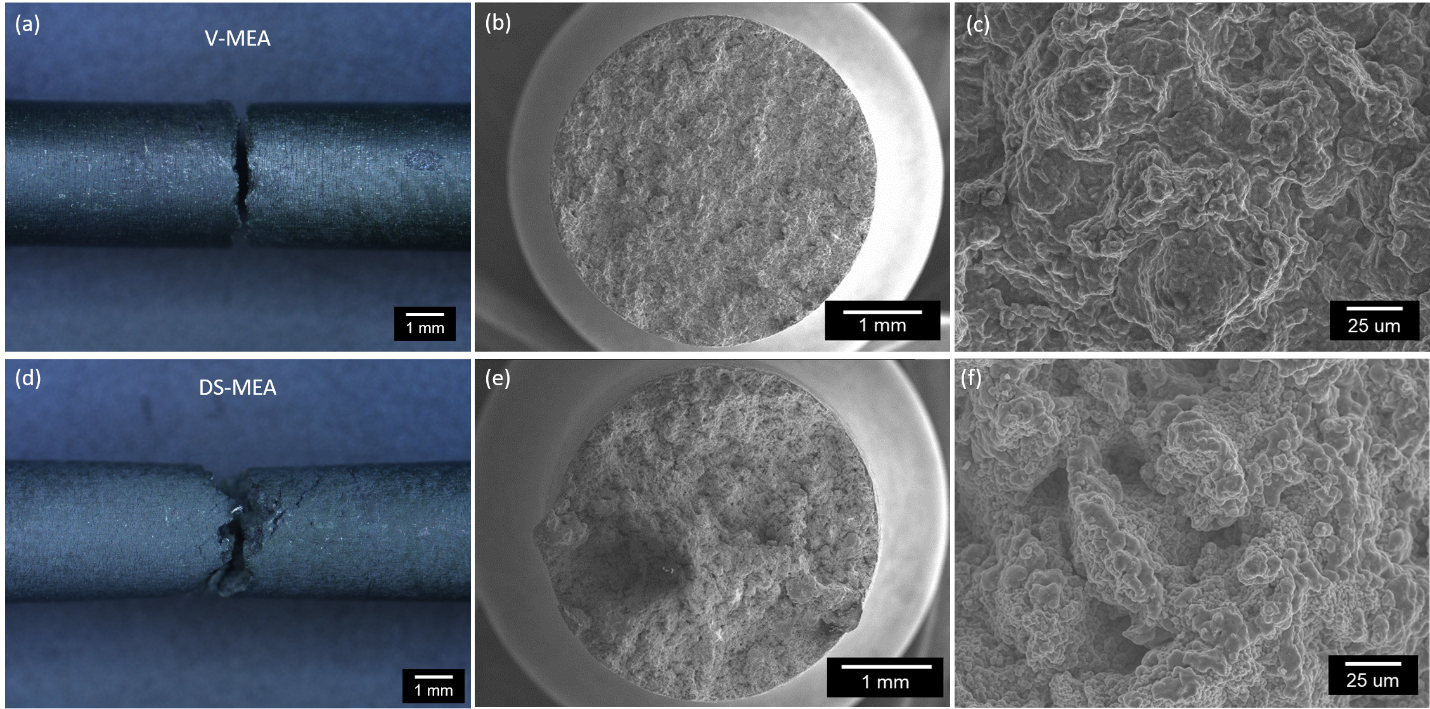


**Supplementary Fig. 4**: **1093C tensile** **fracture surfaces of V-MEA and DS-MEA.** (a) optical and (b-c) SEM images of the fractured V-MEA tensile specimen. (d) optical and (e-f) SEM images of the fractured DS-MEA tensile specimen.
